# First record and description of juvenile stages of *Longidorus artemisiae* Rubtsova, Chizhov & Subbotin, 1999 (Nematoda: Longidoridae) in Poland and new data on *L. juglandicola* Lišková, Robbins & Brown, 1997 based on topotype specimens from Slovakia

**DOI:** 10.1007/s11230-017-9703-y

**Published:** 2017-02-10

**Authors:** Franciszek Wojciech Kornobis, Marek Renčo, Anna Filipiak

**Affiliations:** 1 Department of Zoology, Institute of Plant Protection - National Research Institute, Władysława Węgorka 20, 60-318 Poznan, Poland; 2 Department of Environmental and Plant Parasitology, Institute of Parasitology SAS, Hlinkova 3, 040 01 Košice, Slovak Republic; 3 Department of Biological Pest Control, Institute of Plant Protection - National Research Institute, Władysława Węgorka 20, 60-318 Poznan, Poland

## Abstract

This paper presents the first geographical record of the needle nematode *Longidorus artemisiae* Rubtsova, Chizhov & Subbotin, 1999 outside Russia. This species was found in Poland near the city of Skierniewice in association with nettle (*Urtica dioica* L.). Morphometric and morphological data are provided, including the first description of juveniles of this species. Nematodes of the Polish population differ from the type-population in Russia in possessing a thicker body (lower ‘*a*’ index) in both sexes; males having a longer body and longer spicules; different sex ratio (1:2 in Polish population *vs* 1:1 in the type-population) and somewhat less expanded lips. Molecularly, the Polish population was characterised by sequencing D2-D3 28S rDNA and ITS1 markers. Additionally, new data on these two markers are provided for another species, *Longidorus juglandicola* Lišková, Robbins & Brown, 1997, obtained from topotype specimens from Slovakia. Surprisingly, despite the high morphological similarity of these two species, analysis of their phylogenetic position did not show close phylogenetic relation and several other species (less similar in general morphology) appeared more closely related to both *L. artemisiae* and *L. juglandicola*.

## Introduction

The genus *Longidorus* Micoletzky, 1927 consists of obligatory plant ectoparasites, many of which are of economic importance as plant pests. This importance is further augmented by the fact, that eight species of the genus are known as vectors of viruses of the genus *Nepovirus* (Taylor & Brown, 1997). Additionally, *Longidorus* is rich in species (158 nominal species according to Peneva et al., 2013), which are discriminated mainly on the basis of morphology and morphometrics. However, this approach is complicated by high levels of intraspecific variability in morphometrics and minor interspecific differences leading to substantial overlap among species and increased potential for misidentification (Gutiérrez-Gutiérrez et al., 2013).

To date, 15 species of the genus *Longidorus* have previously been reported from Poland (Kornobis & Peneva, 2011; Kornobis 2012). During a survey of the occurrence of the nematodes of the family Longidoridae in Poland, a previously non-recorded species *Longidorus artemisiae* Rubtsova, Chizhov & Subbotin, 1999 was found. This species was described from Russia on the basis of morphology and morphometrics of adult specimens. Subsequently, Rubtsova et al. (2005) obtained a partial sequence of the D2 domain of 28S rRNA gene from paratype specimens and Subbotin et al. (2014) obtained sequences for D2-D3 domains of 28S rRNA gene and partial sequences of the 18S rRNA gene from other Russian populations and assessed the phylogenetic position of this species. Here we present first record of *L. artemisiae* in Poland. This population was, however, characterised by large intra-specific differences compared to the population from Russia. This paper provides details on the morphology and morphometrics of the Polish population of *L. artemisaie* including the description of juveniles and sequences of D2-D3 domain of 28S rRNA gene and ITS1.

Additionally, differences between the type-population from Russia and the population from Poland made the latter somewhat similar to another species, *Longidorus juglandicola* Lišková, Robbins & Brown, 1997. *Longidorus juglandicola* was described from Slovakia (Lišková et al., 1997) from the rhizosphere of the walnut (*Juglans regia* L.) on the basis of morphology and morphometrics of adult and juvenile specimens. Thereafter records of *L. juglandicola* distribution come only from several localities in Serbia (Barsi & Lamberti, 2002; Krnjaic et al., 2002). As no data on molecular markers of *L. juglandicola* were available, sequences from specimens of the type-population of *L. juglandicola* collected at the type-locality Sorozka (Slovakia) in 2015 are presented together with data on phylogenetic relationships of both species.

## Materials and methods

A total of 925 soil samples were taken during a survey of the occurrence of longidorid nematodes in Poland. In Slovakia, a soil sample was taken from the type-locality of *L. juglandicola* (Sorozka, a hill in eastern Slovakia, see Lišková et al., 1997) in May 2015. The soil sample was taken from the rhizosphere of the same walnut tree (*Juglans regia* L.) in the type-locality. Nematodes were extracted using decant and sieving method (Brown & Boag, 1988), fixation for molecular study in DESS (Yoder et al., 2006); the remaining specimens were heat-killed and fixed in TAF (Courtney et al., 1955). For study of morphology and morphometrics of *L. artemisiae*, specimens were transferred to glycerol as described by Seinhorst (1959). Microscopic slides were made using paraffin ring method and fiber glass to support the coverslip. Observations, measurements and photographs were made using Leica DM5000 microscope. From DESS fixed specimens temporary mounts were made with specimens of both species and photographs illustrating morphology were made (not presented) to retain possibility of checking the morphology of the specimens if necessary. All measurements are in micrometres and are given as the range followed by the means in parentheses.

Subsequently, temporary mounts were dismantled and genomic DNA was isolated from individual specimens using a QIAamp DNA Micro Kit (Qiagen, Hilden, Germany) according to the manufacturer’s a protocol. DNA concentration was measured using a NanoDrop spectrophotometer (Thermo Scientific, Waltham, MA, USA). Amplification and sequencing of the ITS1 rDNA region was performed using primers rDNA2 (Vrain et al., 1992) and rDNA58S (Cherry et al., 1997); amplification of D2-D3 28S rDNA using primers D2A and D3B (Nunn, 1992). PCR conditions were as follows: 95 °C for 5 min, 35 cycles at 94 °C for 45 s, 59 °C for 1 min, 72 °C for 1 min 30 s and a final extension at 72 °C for 10 min (ITS1) and 94 °C for 4 min, 37 cycles at 94 °C for 30 s, 55 °C for 40 s, 72 °C for 1 min and a final extension at 72 °C for 8 min for 28S rDNA. For D2-D3 28S rDNA PCR amplicons were directly sequenced, for ITS1 PCR amplicons were cloned into pCR^TM^4-TOPO^®^vector (TOPO^®^TA Cloning^®^ Kit for Sequencing, Invitrogen) and used to transform into One Shot^®^ TOP10 competent cells for further sequencing. Sequencing was performed by Genomed (Warsaw, Poland).

The newly-generated sequences were subjected to BLAST search to confirm their nematode origins and subsequently deposited under accession numbers KX137849 and KX137850 for D2-D3 28S rDNA of *L. artemisiae* and *L. juglandicola*, respectively. Partial 18S-ITS1-5.8S partial sequences were deposited under numbers KX192397–KX192399, KX192400 and KX192395–KX192396 for *L. artemisiae* and *L. juglandicola*, respectively. Sequences were aligned using ClustalW (Thompson et al., 1994) implemented in MEGA6 (Tamura et al., 2013). For phylogenetic analysis, appropriate base substitution model was chosen using jModelTest 2.1.8 (Darriba et al., 2012) under Akaike information criteria. Sequence dataset was analysed with Bayesian inference (BI) in MrBayes 3.2 (Ronquist et al., 2012) under GTR+I+G model (for both markers). Analysis was initiated from random tree and run with four chains for 500,000 and 1,000,000 generations for D2-D3 28S rDNA and ITS1, respectively. Trees were sampled every 100 generations, ‘burn-in’ samples (25%) were discarded and convergence evaluated, and the remaining samples were retained for further analysis. The topologies were used to generate a 50% majority rule consensus tree. Trees were visualised in FigTree (http://tree.bio.ed.ac.uk/software/figtree/), minor graphic changes were made to improve clearness of data presentation.

**Family Longidoridae Thorne, 1935**

**Genus** ***Longidorus*** **Micoletzky, 1922**

## *Longidorus artemisiae* Rubtsova, Chizhov & Subbotin, 1999

*Host*: *Urtica dioica* L. (*Urticaceae*).

*Locality*: Near the city of Skierniewice (51.9662N, 20.2482E), Poland.

*Prevalence*: Only in 1 out of 925 soil samples.

*Voucher material*: 13 females, 7 males and 23 juveniles were deposited in the collection of the Museum and Institute of Zoology, Polish Academy of Sciences, Warsaw, Poland under accession numbers as follows: females (MIZ 2/2017/10-15; MIZ 2/2017/29-30; MIZ 2/2017/42-44); males (MIZ 2/2017/16-17; MIZ 2/2017/31-32; MIZ 2/2017/35-36; MIZ 2/2017/45); juveniles (MIZ 2/2017/1-9, MIZ 2/2017/18-26/, MIZ 2/2017/37-41). Five male specimens are kept in collection of the first author (FWK).

*Representative DNA sequences*: KX137849 (D2-D3 28S rDNA) and KX192397-9, KX192400 (partial 18S-ITS1-partial 5.8S).

### Description (Figs. 1, 2)

**Fig. 1:**
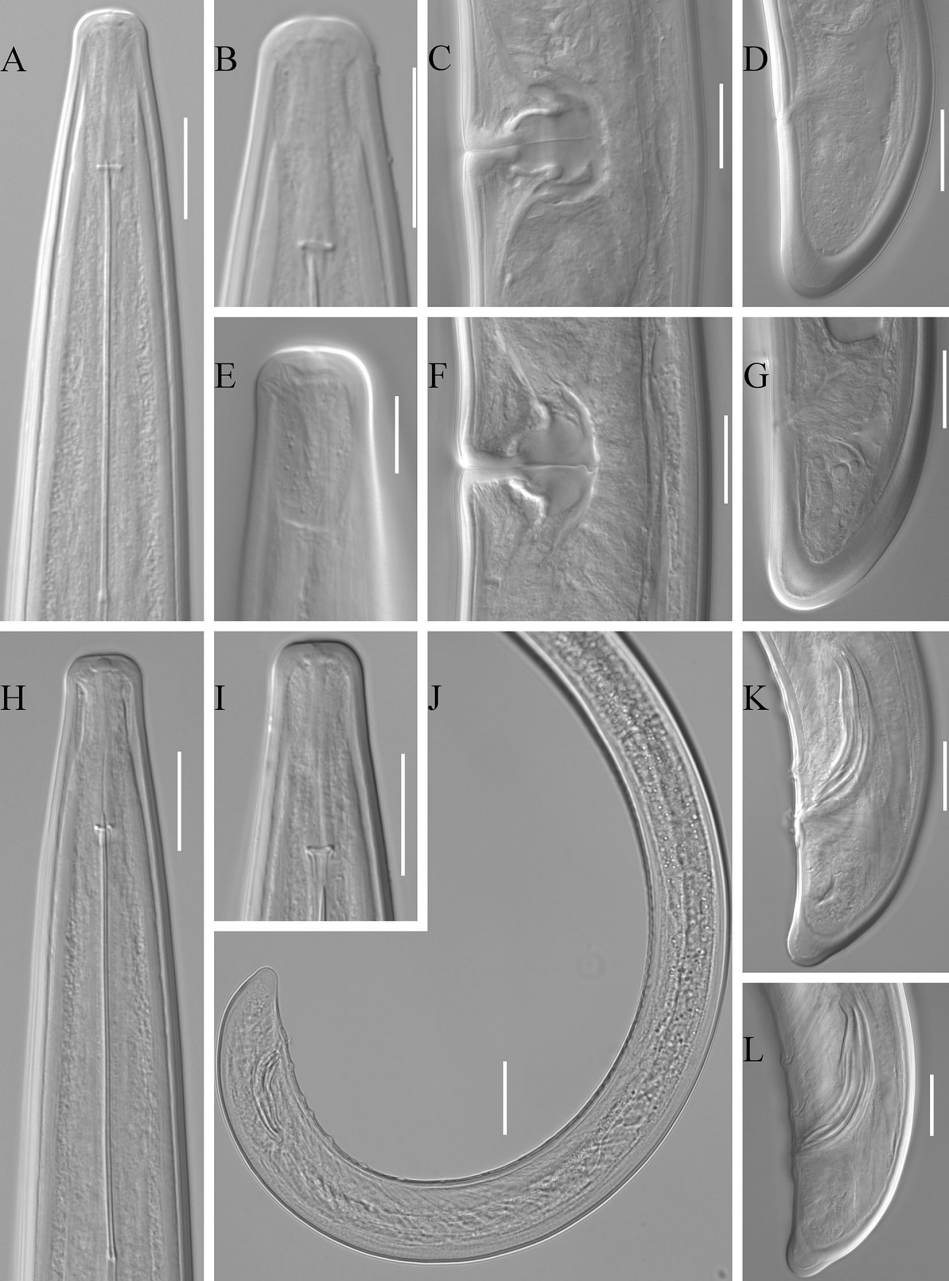
*Longidorus artemisiae* adults. A–G females; H–L males. A, Anterior body; B, Lips; C, F, Vulva; D, G, Tails; E, Amphidial fovea; H, Anterior body; B, Lips; J, Posterior body; K, L, Spicules and tails. *Scale-bars*: A–D, F–I, K–L, 20 µm; E, 10 µm; J, 40 µm

*Female*. [Based on 13 specimens; see also metrical data in Table 1 and Fig. 1A–G). Body C-shaped to open spiral-shaped, more coiled posteriorly. Cuticle with fine, with transverse striations. Cuticle thickness: 3.4–4 at guide ring, 3–4 along most body width, and 7 and 9–10 on ventral and dorsal part of tail. Lips anteriorly flattened to slightly convex, laterally rounded and slightly expanded, set-off from rest of body by almost indistinct to clear constriction. Amphidial fovea pouch-like, protruding to about half of anterior end-guide ring distance, posterior end not bi-lobed. Nerve-ring located at base of odontophore to less than a corresponding body width posterior to it. Pharyngeal bulb occupying about 1/3 of pharynx length. Dorsal and ventro-sublateral gland nuclei 2–2.5 and 3–3.5 wide, respectively. Vagina occupying 46–61 (57.5)% of the corresponding body width, pars distalis and proximalis vaginae 13–15 (4.6) and 15–20 (17.1) long, respectively. Genital tract morphology typical of the genus with sperm cells present in most of examined specimens. Tail bluntly-conoid, ventrally usually flat, sometimes slightly concave or convex; pair of pores present at each side of tail.

**Table 1:** Morphometric data for *Longidorus artemisiae* adults of the population from Poland and the type-population from Russia (Rubtsova et al., 1999)

Locality	Skierniewice (Poland)		Starocherkassk (Rostov region, Russia)	
Host plant	*Urtica dioica* L.		*Artemsia* sp.	
	Females (n = 13)	Males (n = 12)	Females (n = 26)	Males (n = 28)
	Range (mean ± SD)	Range (mean ± SD)	Range (mean ± SD)	Range (mean ± SD)
L	5282–6628 (5833 ± 425)	5630–7039 (6201 ± 393.5)	5100–6500 (5900 ± 100)	4700–6600 (5600 ± 100)
a	86.6–114.3 (100.8 ± 7.6)	104.6–133.6 (118.8 ± 9.9)	109–155 (133 ± 1.9)	113–162 (133 ± 1.7)
b	10.7–17.5 (13.8 ± 1.8)	13.1–16.1 (14.3 ± 0.9)	13.0–21.0 (15.1 ± 0.3)	11.8–17.5 (13.9 ± 0.3)
c	108–165 (134.0 ± 19.7)	118–167 (135.9 ± 15.3)	120–207 (146 ± 3.9)	105–152 (135 ± 2)
c′	0.9–1.4 (1.14 ± 0.13)	1.0–1.3 (1.19 ± 0.12)	1.0–1.6 (1.3 ± 0.03)	1.0–1.6 (1.2 ± 0.02)
d	1.87–2.36 (2.1 ± 0.14)	1.91–2.25 (2.12 ± 0.1)	–	–
d′	1.07–1.68 (1.55 ± 0.16)	1.33–1.57 (1.45 ± 0.07)	–	–
V%	46.5–51.4 (49.2 ± 1.5)	–	46–51 (49 ± 0.3)	–
Odontostyle	80–93 (87 ± 4)	81–91 (86 ± 3)	84–98 (92 ± 1)	90–98 (94 ± 1)
Odontophore	55–69 (61 ± 5) (n = 10)	(55–62) (60 ± 3) (n = 6)	39–50 (41 ± 1)	39–48 (44 ± 1)
Total stylet	139–148 (143 ± 3) (n = 10)	140–151 (146 ± 4) (n = 6)	126–140 (135 ± 1)	132–146 (137 ± 1)
Distance to guide ring	28–33 (31 ± 1)	31–36 (33 ± 2)	27–34 (29 ± 0.3)	28–34 (30 ± 0.3)
Oesophagus length	320–547 (428 ± 53)	401–469 (433 ± 20)	382–464 (412 ± 5)	354–452 (416 ± 6)
Pharyngeal bulb length	111–146 (125 ± 11)	104–127 (115 ± 8)	98–119 (110 ± 2)	99–116 (111 ± 1)
Pharyngeal bulb width	20–23 (22 ± 1)	18–25 (22 ± 2)	19–25 (21 ± 0.4)	17–22 (20 ± 0.3)
Tail length	34–54 (44 ± 5)	35–53 (46 ± 5)	31–46 (40 ± 1)	36–50 (42 ± 1)
Hyaline tip	9–15 (12 ± 2)	10–14 (11 ± 1)	–	–
Prerectum	–	–	232–394 (307 ± 4)	–
Rectum	–	–	23–35 (29)	–
Width at lips	14–16 (15 ± 0)	15–16 (15 ± 1)	14–17 (15 ± 0.2)	14–17 (16 ± 0.2)
Width at guide ring	17–25 (23 ± 2)	20–24 (22 ± 1)	–	–
Width at base of pharynx	44–49 (46 ± 1)	43–47 (45 ± 1)	–	–
Width at vulva in females/midbody in males	53–62 (58 ± 3)	47–56 (52 ± 3)	41–52 (46 ± 1)	38–50 (44 ± 1)
Width at anus	37–41 (39 ± 1)	35–42 (39 ± 2)	28–35 (32 ± 0.4)	32–41 (35 ± 1)
Spicules	–	47–56 (52 ± 3)	–	39–49 (44 ± 1)
Ltgo^a^	4828–6146 (5325 ± 420.4)	5171–6551 (5722 ± 383.3)	4700–6000 (5300 ± 100)	4900–6500 (5300 ± 100)
L/Ltgo	1.07–1.10 (1.08 ± 0.0)	1.07–1.09 (1.08 ± 0.0)	1.08–1.10 (1.09 ± 0.0)	1.07–1.10 (1.08 ± 0.0)

^a^Ltgo, body length–oesophagus length–tail length; *Note* some values from the work of Rubtsova et al. (1999) are rounded

*Male*. [Based on 12 specimens; see also metrical data in Table 1 and Fig. 1H–L). Less frequent than females, sex ratio 1:2. General morphology similar to females, with differences in posterior body part and genital tract. Posterior body part more coiled than in females, tail ventrally concave. Adanal supplements 1–3 pairs, followed by 9–12 single adanal supplements.

**Fig. 2:**
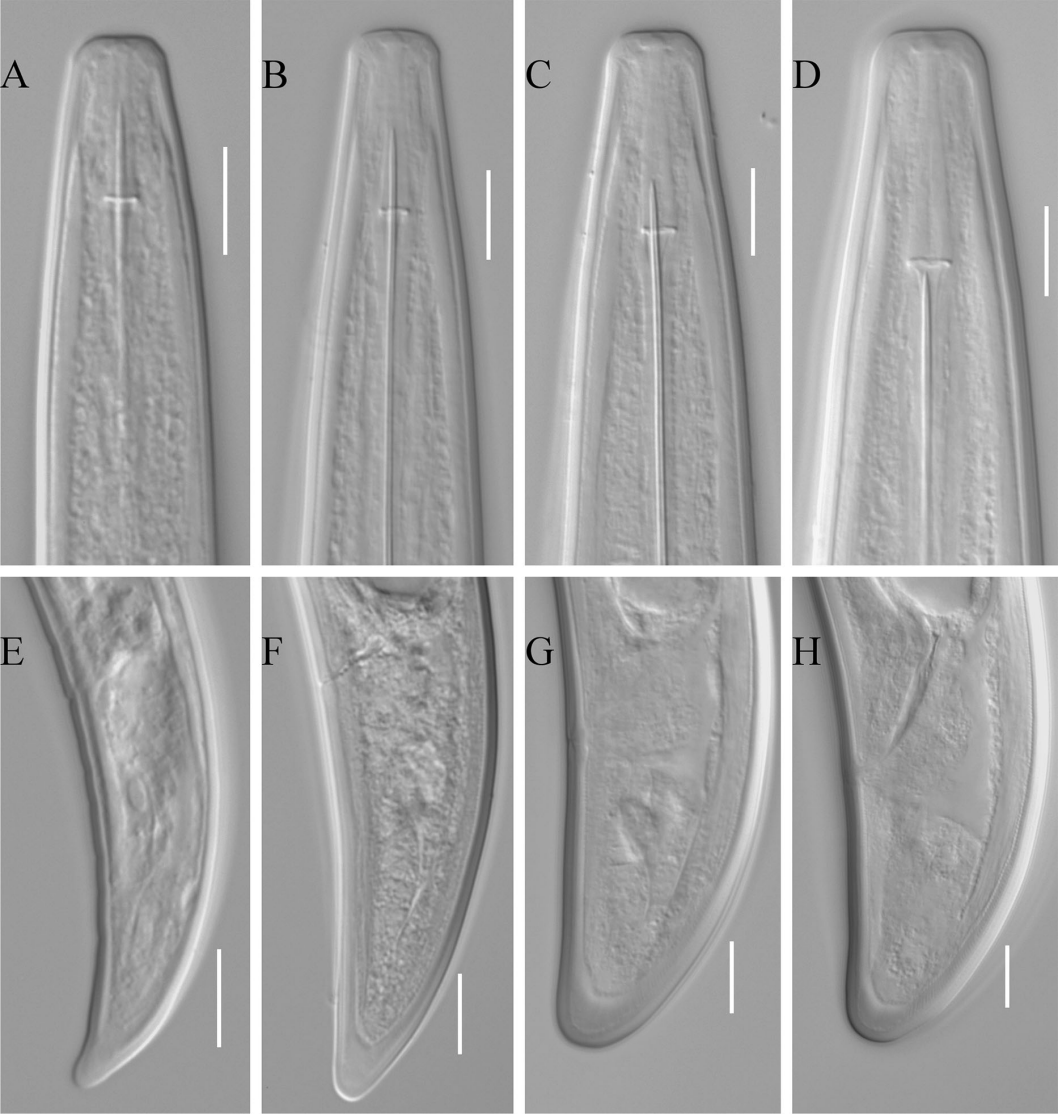
*Longidorus artemisiae* juveniles. A–D, Anterior body of J1–J4, respectively; E–H, tail of J1–J4, respectively. *Scale-bars*: 10 µm

*Juveniles*. [Based on 23 specimens; see also metrical data in Table 2 and Fig. 2A–H). Four developmental stages present. Body from J-shaped to C-shaped in J1, C-shaped in J2, J3 and J4. Shape of lip region in all stages similar to adults, only in J1 lips are not expanded to slightly expanded and not set-off to almost indistinctly set off. Tail shape conoid with a rounded tip in J1 becoming more bluntly rounded in subsequent stages.

**Table 2:** Morphometrics of juvenile *Longidorus artemisiae* of the population from Poland

	J1 (n = 5)	J2 (n = 4)	J3 (n = 4)	J4 (n = 10)
	Range (mean)	Range (mean)	Range (mean)	Range (mean ± SD)
L	1123–1235 (1185)	1827–2070 (1,941)	2340–3170 (2750)	3332–4840 (4134 ± 457)
a	52.7–54.9 (53.8)	64.9–72.4 (69.3)	66.6–90.6 (77.9)	86.9–116.9 (96.2 ± 8.5)
c	24.4–27.1 (25.8)	34–37.5 (35.4)	45–70.4 (59.8)	60.6–102.3 (84.7 ± 11.3)
c′	2.9–3.4 (3.1)	2.5–2.6 (2.6)	1.6–2 (1.8)	1.3–1.8 (1.5 ± 0.2)
Odontostyle	49–52 (50)	53–58 (54)	61–65 (63)	69–78 (74 ± 2)
Replacement odontostyle	52–56 (53)	63–64 (63)	69–80 (73)	80–92 (85 ± 4)
Tail length	43–51 (46)	53–58 (55)	44–52 (46)	45–55 (49 ± 4)
Width at lips	8–9 (8)	10–11 (10)	11–12 (12)	11–14 (13 ± 1)
Width at mid-body	22–23 (22)	27–30 (28)	35–37 (35)	38–46 (43 ± 2)
Width at anus	15–16 (15)	20–23 (21)	26–28 (27)	31–36 (33 ± 2)

## **Phylogenetic position of*L. artemisiae*and*L. juglandicola***

The newly-generated sequences of D2-D3 28S rDNA and sequences containing partial 18S, whole ITS1 region and partial 5.8S are listed together with their GenBank accession numbers in Table 3 and 4. The D2-D3 28S rDNA sequence of the Polish isolate of *L. artemisiae* was 741 bp long. BLAST search revealed maximum scores with four Russian sequences for *L. artemisiae* (KF242313–KF242316, 98–99% similarity). Next sequences with highest scores were those for *L. uroshis* Krnjaić, Lamberti, Krnjaić, Agostinelli & Radicci, 2000 from Slovakia (EF538754, 96% of similarity). The D2-D3 sequence of *L. juglandicola* was 716 bp long. Results with maximum BLAST scores included a sequence of *Longidorus* sp. (KF242335) (96% of similarity). These sequences and several other with subsequent highest BLAST scores (listed in Table 3) were used for phylogenetic reconstruction.

**Table 3:** List of species used in the analyses and GenBank accession numbers for D2-D3 28S rDNA sequences

Species	Accession number	Reference
*L. artemisiae* Rubtsova, Chizhov & Subbotin, 1999	KF242313–6	Subbotin et al. (2014)
	KX137849	Present study
*L. attenuatus* Hooper, 1961	AY601572	He et al. (2005)
	KR911851	Kornobis et al. (2016)
*L. carpathicus* Lišková, Robbins & Brown, 1997	AF480072	Rubtsova et al., (2001)
*L. closelongatus* Stoyanov, 1964	KJ802863	Tzortzakakis et al. (2014)
	KJ802866	
*L. dunensis* Brinkman, Loof & Barbez, 1987	AY593056–7	Unpublished
*L. elongatus* (De Man, 1876) Thorne & Swanger, 1936	AF480076	Rubtsova et al. (2001)
	KF242305–6	Subbotin et al. (2014)
*L. euonymus* Mali & Hooper, 1973	KF242331–3	Subbotin et al. (2014)
*L. juglandicola* Lišková, Robbins & Brown, 1997	KX137850	Present study
*L. iranicus* Sturhan & Barooti, 1983	KP222294	Maafi et al. (2015)
*L. intermedius* Kozłowska & Seinhorst, 1979	KT308868	Archidona-Yuste et al. (2016)
	KF242311–2	Subbotin et al. (2014)
	AF480074	Rubtsova at al. (2001)
	AY593058	Holterman et al. (unpublished)
*L. kuiperi* Brinkman, Loof & Barbez, 1987	AM911623	De Luca et al. (2009)
*L. moesicus* Lamberti, Choleva & Agostinelli, 1983^a^	KJ802874; KJ802876^a^	Tzortzakakis et al. (2014)
*L. piceicola* Lišková, Robbins & Brown, 1997	AY601577	He at al. (2005)
*L. uroshis* Krnjaić, Lamberti, Krnjaić, Agostinelli & Radicci, 2000	EF538754	Kumari et al. (2009)
*Longidorus* sp. 3	KF242335	Subbotin et al. (2014)
*Longidorus* sp. 4	KF242334	Subbotin et al. (2014)
*Xiphinema diversicaudatum* (Micoletzky, 1927) Thorne, 1939	KF292280	Subbotin et al. (2014)

^a^Sequences KJ802874 and KJ802876 are described in GenBank as *L. moesicus* and to avoid confusion we have used this name here. However, *L. moesicus* has been synonimised with *Longidorus iranicus* Sturhan & Barooti, 1983 by Maafi et al. (2015)

**Table 4:** List of species used in the analyses and GenBank accession numbers for ITS1 sequences

Species	Accession number	Reference
*L. artemisiae* Rubtsova, Chizhov & Subbotin, 1999	KX192397–9	Present study
	KX192400	
*L. crassus* Thorne, 1974	AF511414	Ye et al. (2004)
*L. cretensis* Tzortzakakis, Peneva, Terzakis, Neilson & Brown, 2001	KJ802892	Tzortzakakis et al. (2014)
*L. elongatus* (De Man, 1876) Thorne & Swanger, 1936	AF511417	Ye et al. (2004)
	AJ549986–7	De Luca et al. (2004)
	GU199044	Pedram et al. (2010)
*L. fragilis* Thorne, 1974	AF511418	Ye et al. (2004)
*L. grandis* Ye & Robbins, 2003	AF511419	Ye et al. (2004)
*L. iranicus* Sturhan & Barooti, 1983	KP222295	Maafi et al. (2015)
*L. juglandicola* Lišková, Robbins & Brown, 1997	KX192395–6	Present study
*L. moesicus* Lamberti, Choleva & Agostinelli, 1983	KJ802893	Tzortzakakis et al. (2014)
*L. orientalis* Loof, 1982	KP406947	Subbotin et al. (2015)
*L. perangustus* Roshan-Bakhsh, Pourjam & Pedram, 2016	KT593863	Roshan-Bakhsh et al. (2016)
*L. pseudoelongatus* Altherr, 1976	KJ802894	Tzortzakakis et al. (2014)
*Longidorus* sp. M30	FJ009679	Niknam et al. (2010)
*L. sturhani* Rubtsova, Subbotin, Brown & Moens, 2001	FJ009680	Niknam et al. (2010)
*Xiphinema index* Thorne & Allen, 1950	AY430175	He et al. (unpublished)

**Fig. 3:**
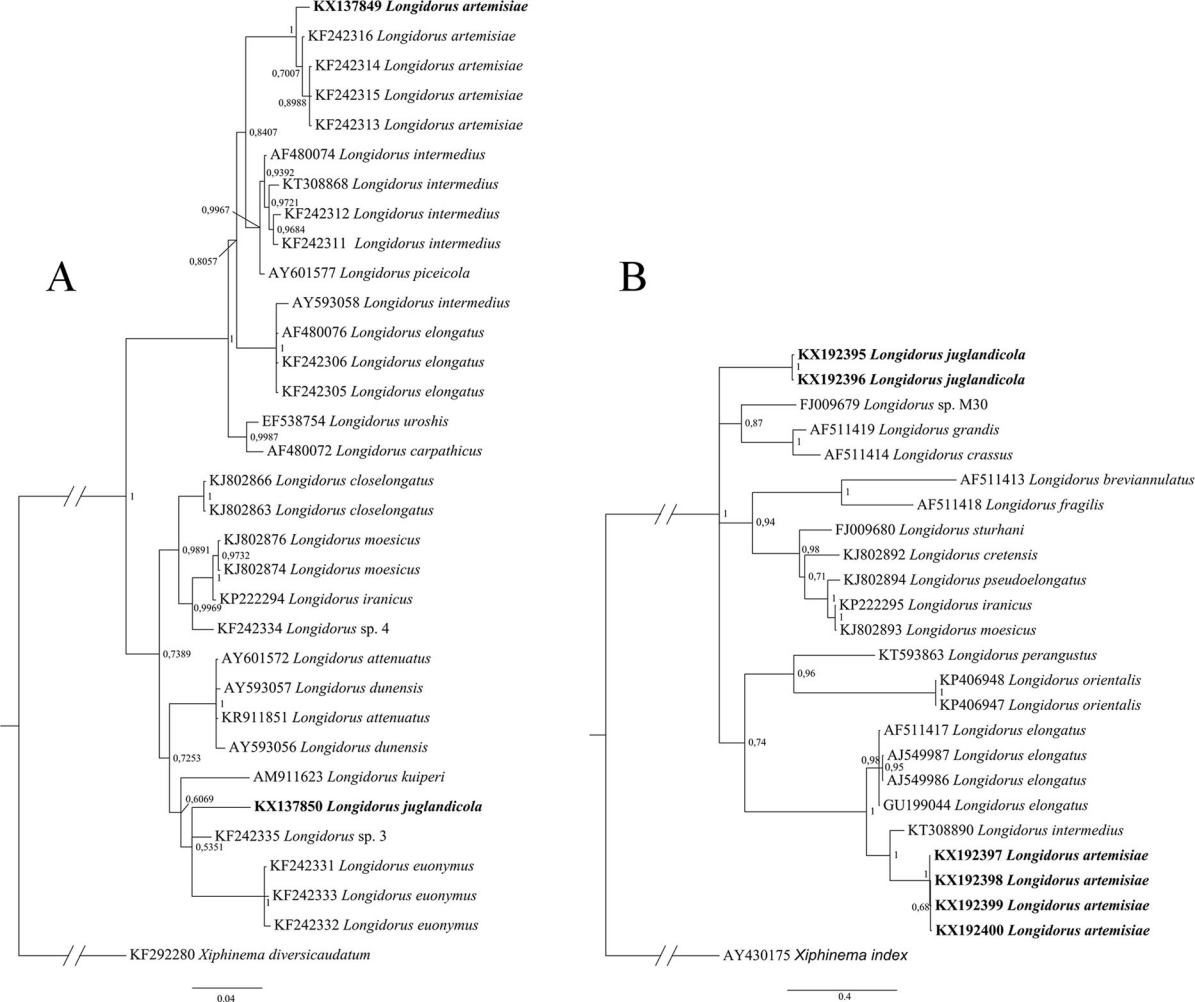
Bayesian inference phylogenetic trees including *L. artemisiae* and *L. juglandicola* (sequences indicated in bold) based on: A, D2-D3 28S rDNA marker; B, ITS1

In the ITS1 region-containing sequences, four sequence variants were recovered for *L. artemisiae* and two for *L. juglandicola*. These sequences were trimmed to include ITS1 only and used for both BLAST search and phylogeny reconstruction. BLAST search of sequences did not show exact matches with sequences of neither *L. artemisiae* nor *L. juglandicola* from this study. Sequences of *L. artemisiae* revealed 80–84% similarity with the sequences for *L. elongatus* (De Man, 1876) Thorne & Swanger, 1936 and *L. intermedius* Kozłowska & Seinhorst, 1979 (accession numbers: AF511417, AJ549986–AJ549987, GU199044 and KT308890). Other sequences were characterised by very low query coverage, resulting in low total scores of BLAST searches. None of sequences available in the GenBank database was significantly similar to sequences obtained in this study for the type-population of *L. juglandicola* from Slovakia. To reconstruct the phylogeny, ten first results from BLAST searches were included to the alignment (some sequences repeated in both searches). The final list of ITS1 sequences used for phylogeny reconstruction is presented in Table 4. The final alignment of the D2-D3 28S rDNA sequences contained 689 positions and the alignment of ITS1 sequences comprised 896 positions. The phylogenetic trees are presented on Fig. 3 A and B. Some relationships were unresolved, however results from both D2-D3 28S rDNA and ITS1 sequences enable two conclusions. First, *L. artemisiae* as well as *L. juglandicola* formed their own clades, well distinguishable from the other species in this analysis. Secondly, these two species are not closely related, as many other species appear more closely related to each of them.

## Discussion

Morphology and morphometrics of the population of *L. artemisiae* from Poland studied here revealed several differences in comparison with the original description by Rubtsova et al. (1999). These include a more slender body in the specimens from the type-population (‘a’ index value of 109–155 (133 ± 1.9) and 113–162 (133 ± 1.7) *vs* 86.6–114.3 (100.8 ± 7.6) and 104.6-133.6 (118.8 ± 9.91) in females and males, respectively. Low ‘a’ index can sometimes be a result of inappropriate preparation of microscopic slides, where specimen is flattened by coverslip. Here microscopic slides were made with care and using glass fibre (see ‘Materials and methods’) Nevertheless, we have prepared additional slides which additional specimens. On these slides pieces of broken coverslip (130–170 µm thick according to the manufacturer) were used instead of glass fibres. Measuring body width of several specimens from such slides did not reveal any important differences compared to values given in Table 1 (data not shown). Other differences worth mentioning include: longer body of male specimens from Poland, 6,201 ± 393.5 *vs* 5,600 ± 100 µm for the Russian type-population; and longer spicules in specimens of the Polish population compared with the type-population: 39–49 (44 ± 1) *vs* 47–56 (52 ± 3) µm. Additionally, the population from Poland was characterised by a different sex ratio (1:2 *vs* 1:1 in the type-population) and the specimens possess lips somewhat less expanded than in the population described by Rubtsova et al. (1999). There was a large difference in odontophore length (see data in Table 1), however this result is not comparable because in the description of Rubtsova et al. (1999) the odontophore was measured from its junction with odontostyle to the posterior end of the ventral sinus while in the present study we measured the entire odontophore.

According to Rubtsova et al. (1999), the code for identifying *L. artemisiae* in the polytomous key of Chen et al. (1997) is A3-B23-C23-D3-E1-F3-G23-H2-I2. Despite the differences mentioned above, the population of this species from Poland fits into that code and no changes are required. This code is rather similar to that of *L. juglandicola* which, according to Chen et al. (1997) is A23-B34-C3-D23-E2-F34-G23-H12-I2.

*Longidorus artemisiae* was described from the rhizosphere of *Artemisia* sp. (see Rubtsova et al., 1999). Subsequently, Subbotin et al. (2014) reported three populations associated with *Elytrigia* sp., *Poa* sp. and *Trifolium* sp. The present finding of this species in the rhizosphere of nettle in Poland extends the known host plant list of this nematode as well its geographical range.

Similarly to the results obtained by Subbotin et al. (2014), *L. artemisiae* was most closely related to *L. intermedius* and *L. piceicola* based on D2-D3 28S rDNA (Fig. 3A). No comparative data are available for the ITS1 marker of *L. artemisiae* and any markers of *L. juglandicola.* The fact that this two species are not closely related was not expected taking into account that *L. artemisiae* and *L. juglandicola* are rather similar in terms of general morphology and morphometrics. For example, this similarity is well visible in the code of Chen et al. (1997). Despite that, many other species which are less similar in general morphology, appear to be more closely related to both *L. artemisiae* and *L. juglandicola* (Fig. 3A, B).
